# International Clinics

**Published:** 1902-04

**Authors:** 


INTERNATIONAL CLINICS.—A quarterly of clinical lectures and especially prepared articles of medicine, neurology, surgery, therapeutics, obstetrics, pediatrics, pathology, dermatology, diseases of the eye, ear, nose and throat, and other topics of interest to students and practitioners. By leading members of the medical profession throughout the world. Edited by Henry W. Cattell, A. M., M. D., Philadelphia, with the colaboration of John B. Murphy, M. D., of Chicago; Alex D. Blockader, M. D., of Montreal; H. C. Wood, M. D., of Philadelphia; T. M. Rotch, M. D., of Boston; E. Landolt, M. D., of London; Thos. G. Morton, M. D., of Philadelphia; C. H. Reed, M. D., of Philadelphia; J. W. Ballantyne, M. D., of Edenburg, and John Harold, M. D., of London. With regular correspondents in Montreal, London, Paris, Leipsig, and Vienna. Vol. IV; tenth series, 1901. Price, $2.00 per volume. Philadelphia: J. B. Lippincott Company. 1901.

In this most interesting volume, under the head of "Therapeutics," Dr. H. C. Wood contributes an article about the United States pharmacopœia; Dr. A. Potain writes on the "Indications and Contraindications for the Use of Digitalis in Treating Heart Diseases"; Dr. Douglass Graham on "Massage in Raynaud's Disease"; Professor B. Grassi on the "Mosquitos and the Prophylaxis of Malaria." There is also found a symposium on genito-urinary diseases by A. Renault, M. D.; James Redersen, M. D.; Felix Guyon, M. D.; A. H. Ohmann-Dumenil, M. D., and A. Fournier, M. D. Under the head of "General Medicine" the following subjects are discussed. Recent advances in diagnosis, cases presenting renal, cardiac and pulmonary lesions; aortic and mitral regurgitation; a service in the medical out-patient department of the Pennsylvania Hospital; observations on the phlebitis of advanced phthisis. Similar practical articles appear under the headings of pathology, surgery, neurology, laboratory methods, etc. T. J. B.

